# Magnetic resonance imaging and previous cesarean section in placenta accrete spectrum disorder: Predictor model

**DOI:** 10.1016/j.clinsp.2022.100027

**Published:** 2022-03-29

**Authors:** Rodrigo Pamplona Polizio, Fernando Ide Yamauchi, Renata Franco Pimentel Mendes, Stela Verzinhasse Peres, Mario Macoto Kondo, Rossana Pulcineli Vieira Francisco

**Affiliations:** a Departamento de Radiologia e Diagnóstico por Imagem do Hospital das Clínicas da Faculdade de Medicina da Universidade de São Paulo (HCFMUSP), São Paulo, SP, Brazil; b Departamento de Obstetrícia e Ginecologia do Hospital das Clínicas da Faculdade de Medicina da Universidade de São Paulo (HCFMUSP), São Paulo, SP, Brazil

**Keywords:** Placenta accrete, Placenta previa, Cesarean section, Magnetic resonance imaging

## Abstract

**Objective:**

To evaluate objective criteria of Magnetic Resonance Imaging (MRI) of Placenta Accreta Spectrum disorder (PAS) analyzing interobserver agreement and to derive a model including imaging and clinical variables to predict PAS.

**Methods:**

A retrospective review including patients submitted to MRI with suspicious findings of PAS on ultrasound. Exclusion criteria were lack of pathology or surgical information and missing or poor-quality MRI. Two radiologists analyzed six MRI features, and significant clinical data were also recorded. PAS confirmed on pathology or during intraoperative findings were considered positive for the primary outcome. Variables were tested through logistic regression models.

**Results:**

Final study included 96 patients with a mean age of 33 years and 73.0% of previous C-sections. All MRI features were significantly associated with PAS for both readers. After logistic regression fit, including MRI signs with a moderate or higher interobserver agreement, intraplacental T2 dark band was the most significant radiologic criteria, and ROC analysis resulted in an AUC = 0.782. After including the most relevant clinical data (previous C-section) to the model, the ROC analysis improved to an AUC = 0.893.

**Conclusion:**

Simplified objective criteria on MRI, including intraplacental T2 dark band associated with clinical information of previous C-sections, had the highest accuracy and was used for a predictive model of PAS.

## Highlights

• Predicting placenta accreta spectrum disorder with magnetic resonance imaging.
• Estimating probability of placenta accreta spectrum disorder.

## Introduction

The Placenta Accreta Spectrum disorder (PAS) encompasses previous terms such as morbidly adherent placenta, placental invasion, and abnormally invasive place,[1] and is the most accepted term used in the clinical practice to standardize the terminology and is included in the Federation of Gynecology and Obstetrics (FIGO) consensus guidelines.

The most common risk factors for PAS are prior cesarean delivery and placenta previa, with rising incidence in the past years.[2,3] Moreover, the risk of PAS is progressively increased with each Cesarean section (C-section),[4] with a likelihood of 11% of PAS in a patient with a single prior C-section and 61% in women with three prior C-sections.[5,6] Other risk factors include increasing maternal age and history of curettage or other uterine surgery.[3] PAS is associated with maternal morbidity and can lead to intrapartum hemorrhage and is a life-threatening situation for the mother and the fetus,[7,8] and the prenatal diagnosis of PAS is associated with reduction of these complications,[9,10] indicating its significative importance.

Ultrasound is the most widely used method for the diagnosis during the prenatal period given wide availability and relatively low-cost method and has a good accuracy mainly for the anterior uterine wall evaluation. Besides that, ultrasound allows a longitudinal assessment of women at risk for PAS increasing its possibility to reach the final diagnosis.[10] Magnetic Resonance Imaging (MRI) is usually performed only once as a secondary tool in cases in which ultrasound is inconclusive,[10] mainly for the evaluation of the posterior uterine wall[7,11, 12, 13, 14, 15] and improves surgical planning by identifying the location of invasive placentation.[16] These different methods have already been compared and showed good overall diagnostic accuracy in detecting PAS disorders with some differences between them.[17]

Several studies have evaluated MRI criteria associated with PAS; however, most articles evaluated isolated criteria, had smaller sample sizes, or did not have pathological and surgical confirmation.[1,18, 19, 20, 21, 22, 23, 24, 25] Moreover, the interpretation of the impact of each MRI sign to the final diagnosis is not well understood as well there is a lack of evidence on how to stratify the surgical risk of women affected by PAS.[26] Therefore, the objective of this study was to evaluate the objective criteria of MRI for the diagnosis of PAS, correlating to intraoperative findings and pathology and then creating a model to predict PAS, including imaging and clinical variables.

## Materials and methods

### Subjects

**Fig. 1:**
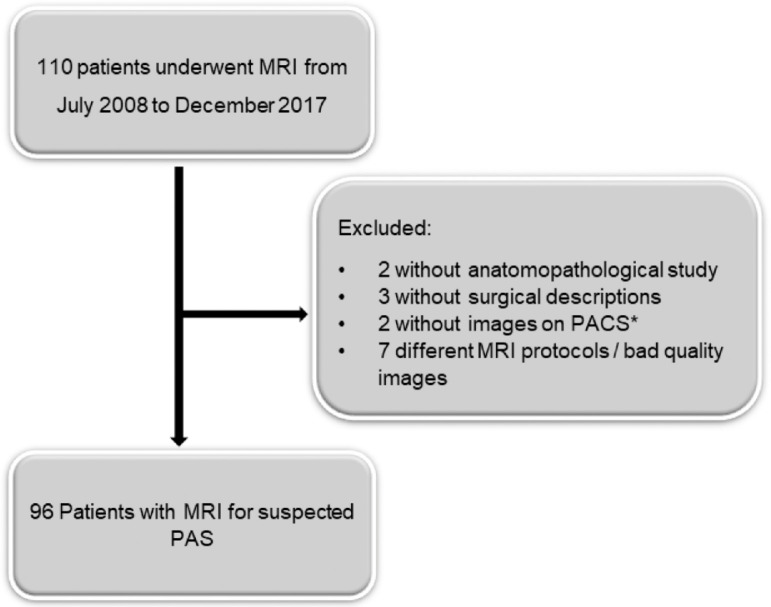
Flowchart of the study population. *PACS (Picture Archiving and Communication System).

The Institutional Review Board (IRB) approved this retrospective study and granted a waiver of consent. This is a tertiary hospital, and the obstetric department is a reference for high-risk patients. Patients that are diagnosticated by ultrasound with placenta previa and have the suspicion of PAS are routinely sent to MRI. Therefore, the authors included in the study pregnant women who performed aj MRI under those circumstances during the period from July 2008 to December 2017. At last, the authors had a total of 110 patients, but 14 patients were not included in the analysis for different reasons, such as an anatomopathological study of the placenta not available (n = 2), surgical description not available (n = 3), MRI images not available (n = 2) and low-quality MRI images (n = 7) (Fig. 1).

The following clinical data were also recorded: maternal age, gestational age at MRI, gestational age at delivery, the time between MRI and delivery, ethnic group, parity, previous C-sections, abortions, history of curettage, and other uterine surgeries. PAS confirmed on the pathology of surgical specimen and/or signs of PAS during intraoperative findings were considered as positive for the primary outcome (Table 1).

**Table 1:** Characteristics of study subjects.

Clinical data	Mean ± SD or n (%)
Maternal Age (years)	33.65 ± 5.39
Gestational age at MRI (weeks)	32.20 ± 3.79
Gestational age at delivery (weeks)	35.70 ± 2.68
Race	
- White	77 (80.2%)
- Nonwhite	16 (16.7%)
- Uninformed	3 (3.10%)
Parity	
- 1	11 (11.5%)
- 2	21 (21.9%)
- 3	18 (18.8%)
- 4	22 (22.9%)
- ≥5	24 (25.0%)
Abortions	
- 0	53 (55.2%)
- 1	28 (29.2%)
- 2	10 (10.4%)
- 3	5 (5.20%)
Previous C-section	
- 0	27 (28.1%)
- 1	33 (34.4%)
- 2	18 (18.8%)
- 3	15 (15.6%)
- 4	3 (3.10%)
Other surgeries (myomectomy)	
- 0	93 (96.6%)
- 1	3 (3.10%)
Curettage	
- 0	63 (66.6%)
- 1	23 (24.0%)
- 2	5 (5.20%)
- 3	5 (5.20%)

SD, Standard Deviation; n, number.

### Image acquisition

All MRI examinations were performed on a 1.5T unit (Signa HD × TM, General Electric Healthcare) with body array coils, including axial, coronal, and sagittal T2-weighted Single Shot Fast Spin-Echo (SS-FSE); axial, coronal, and sagittal balanced steady-state free precession (Fast Imaging Employing Steady-State Acquisition – FIESTA); axial and sagittal fat-suppressed ultra-fast spoiled gradient-echo T1-weighted (LAVA) without intravenous contrast media.

The parameters for SS-FSE images were Repetition Time (TR)/Echo Time (TE), 650–16000 / 50–90 msec; Flip Angles (FA), 90; Field of View (FOV), 120–480 mm; slice thickness, 4.5 mm. For balanced ‒ steady-state free-precession ‒ FIESTA images, they were TR/TE, 3800–6000/ 50–90 msec; FA = 90; FOV, 120–480 mm; slice thickness, 4.5 mm, and for T1 LAVA images, they were: TR/TE, 200–230 / 1.8‒11 msec; FA = 80; FOV, 340–380 mm; slice thickness, 4.5 mm.

### Image interpretation

Two board-certified blinded radiologists with different levels of expertise (2 and 8 years of experience in abdominal radiology) retrospectively analyzed the exams on a Likert scale^1^, ^2^, ^3^, ^4^, ^5^ for each sign of PAS.

**Fig. 2:**
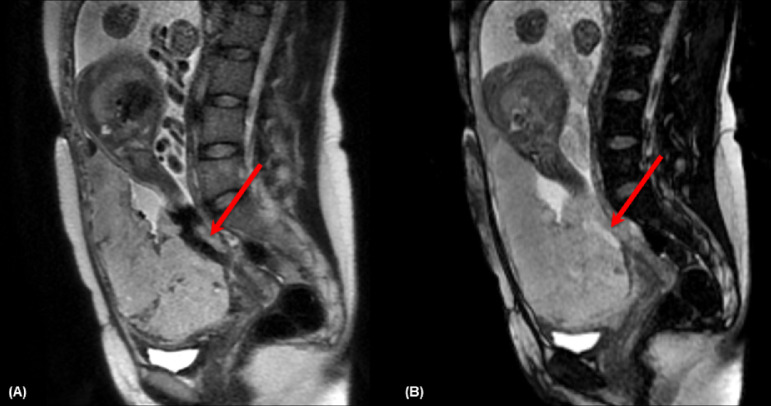
28-year-old woman at 34 weeks' gestation with two previous C-sections. (A) Sagittal T2 weighted image shows intraplacental abnormal vascularity with a flow void in the placenta (arrow), and on (B) axial FIESTA there is a high signal on the structure indicating the presence of vascular flow.
 - 5: > 6mm
 - 4: 5-6 mm
 - 3: 3-4 mm
 - 2: 1-2 mm
 - 1: absent
Intraplacental T2 dark band: wedge-shaped areas of low signal intensity on T2-weighted images and FIESTA (19) (Fig. 3, Fig. 4).

The MRI signs used for evaluation were:

1- Intraplacental abnormal vascularity: tortuous enlarged flow voids observed on T2 and corresponding high signal on FIESTA, indicating vascular flow[18] (Fig. 2).

**Fig. 3:**
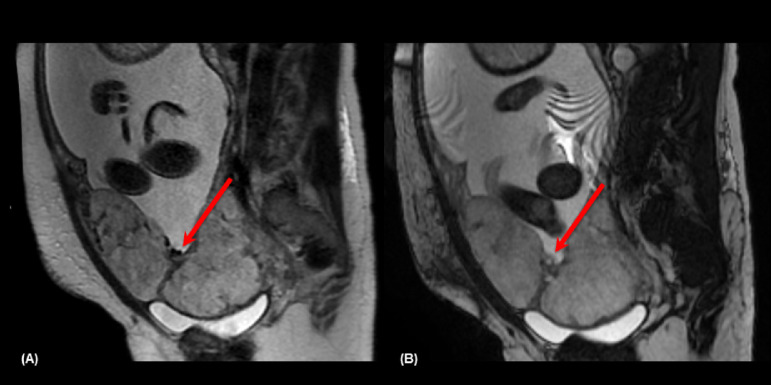
34-year-old woman at 36 weeks' gestation and one previous C-section. (A) Sagittal T2 weighted image shows intraplacental T2 dark band (arrow), and on (B) sagittal FIESTA the band remains with the low signal intensity.

**Fig. 4:**
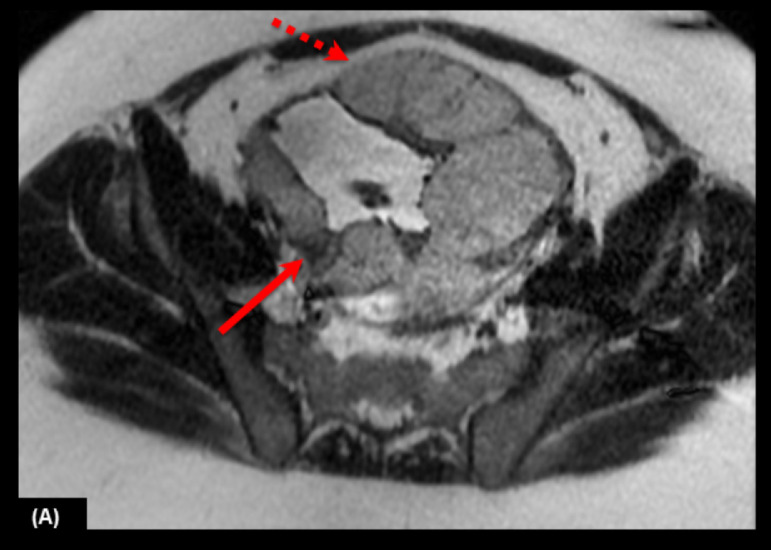
42-year-old woman at 35 weeks' gestation and one previous C-section. (A) Axial T2 weighted images shows intraplacental T2 dark band (arrow) and placental bulges (dashed arrows).
 - 5: > 20 mm
 - 4: 15-20 mm
 - 3: 10-14 mm
 - 2: < 10 mm
 - 1: absent
2- Placental bulge: focal bulging of the uterine contour.[14,19,24,27]
 - 5: clear loss of myometrial congruency or invasion of adjacent organs
 - 4: focal bulges
 - 3: disruption of the normal pear shape of the uterus with the lower uterine segment being wider than the fundus
 - 2: slight irregularities on uterine contours
 - 1: normal contours
3- Placental protrusion sign: placenta pushing inferior to the internal os.[21]
 - 5: > 15 mm
 - 4: 11-15 mm
 - 3: 6-10 mm
 - 2: < 5 mm
 - 1: absent
4- Myometrial thinning: focal thinning and indistinctness of the myometrium and loss of thin dark uteroplacental interface on T2-weighted images.[18,28,29]
5- Heterogeneous placenta: marked heterogeneous intensity within the placenta (Fig. 5).[18,30, 31, 32]

**Fig. 5:**
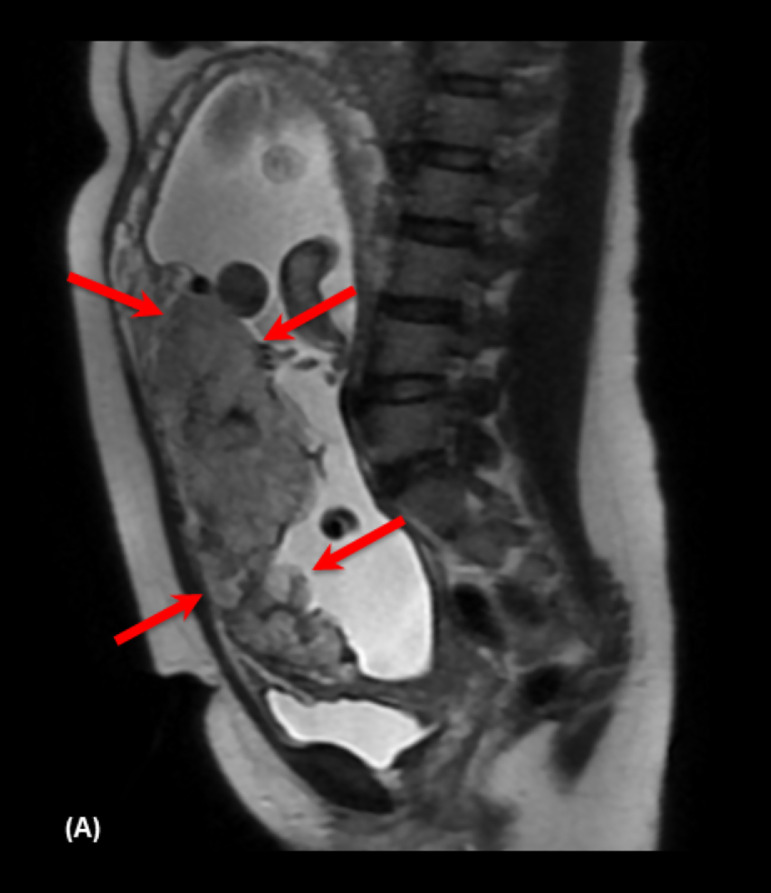
35-year-old woman at 35 weeks' gestation and two previous C-sections. (A) Sagittal T2 weighted image shows a heterogeneous placenta (arrows).

### Statistical analysis

#### Pathological and surgical data

The surgical description had three outcomes: normal discharge of the placenta, focal attachment (small bleeding during placental discharge but no clinical relevance/no need for blood transfusions or other intervention), and hysterectomy. Only hysterectomy was considered positive for PAS. For pathological analyses, there were five outcomes: normal placenta, focal acretism, accreta, increta and percreta. Normal placenta and focal acretism were considered negative for pathological PAS.

#### Clinical and imaging analyses

Descriptive analyses of the data were performed by using absolute and relative frequencies, central tendency, and dispersion measures. The comparison between the groups of PAS and no PAS was made with a Mann-Whitney test for non-parametrical variables and *t-*Student test for parametrical variables.

The interobserver variability of all MRI signs was assessed using the Kappa (κ) value. A κ-value of ≤ 0.20 was interpreted as slight agreement, 0.21–0.40 fair agreement, 0.41–0.60 as moderate agreement, 0.61–0.80 as substantial agreement, and ≥ 0.81 as almost perfect agreement.[33]

For the development of the predictor model, both readers reassessed the discordant criteria and reviewed it in consensus. This strategy was made to extract the best possible information from the MRI and build the best predictor model possible. To identify which MRI signs and clinical variables were most significant, analyses through multiple binary logistic regression models were made in other to identify its coefficients, Odds Ratios (OR), and their respective 95% Confidence Intervals (95% CI). The significant variables (p < 0.005) were tested on the multiple modeling and those which presented a value of p < 0.20 by the Stepwise technique.

Receiver Operating Characteristic (ROC) analysis was used for evaluation of the diagnostic performance using only the most significant MRI sign and using the most significant MRI sign combined with the most significant clinical data for PAS. The areas under the ROC Curves (AUC) were estimated nonparametrically for non-ordinal score assessments.

Analyses were conducted using statistical software (Statistical Package for the Social Science – SPSS, version 20.0 for Windows).

## Results

The final sample of 96 patients was divided into two groups according to anatomopathological study and/or surgical descriptions: patients with PAS (n=42) and patients without PAS (n=54) (Table 1).

### Clinical variables

For all the clinical variables analyzed, only parity and previous C-sections showed significant differences between the PAS and no PAS groups. All other variables: abortions, curettage, maternal age, gestational age at MRI, gestational age at delivery, and time interval between MRI and delivery showed no significant differences between the PAS and no PAS groups (Table 2).

**Table 2:** Analyses of clinical data and association with placental acretism.

Clinical variables	PAS	No PAS	p
Maternal Age (years), mean (SD)	33.7 (± 5.4)	32.9 (± 5.6)	0.486^a^
Gestational age at MRI (weeks), mean (SD)	31.5 (± 4.7)	32.6 (± 2.82)	0.194^a^
Gestational age at delivery (weeks), mean (SD)	35.2 (± 3.2)	36.1 (± 2.0)	0.114^a^
Time interval between MRI and delivery (weeks), mean (SD)	3.6 (± 3.4)	3.4 (± 2.4)	0.803^a^
Parity, median (min-max)	2 (0‒8)	1 (0‒5)	<0.0001^b^
Previous C-Section, median (min‒max)	2 (0‒4)	1 (0‒3)	<0.0001^b^
Abortions, median (min‒max)	0 (0‒3)	0 (0‒3)	0.464^b^
Curettage, median (min‒max)	0 (0‒3)	0 (0‒3)	0.061^b^

SD, Standard Deviation; min, minimum; max, maximum.

a *t*-Student test.

b Mann-Whitney test.

### Differences in each MRI sign between PAS and no PAS groups and diagnostic performance

All MRI signs (intraplacental abnormal vascularity, Intraplacental T2 dark band, placental bulge, placental protrusion sign, myometrial thinning, and heterogeneous placenta) showed significant differences between the PAS and no PAS groups for both reader A and reader B (Table 3).

**Table 3:** MRI feature analyses and performance for each radiologist.

	PAS median (min‒max)	No PAS median (min‒max)	p*	SEN	SPE	PPV	NPV
**Radiologist A**							
Intraplacental T2 dark band	5 (1‒5)	1 (1‒5)	<0.0001	73.8%	74.1%	68.9%	78.4%
Abnormal vascularity	5 (1‒5)	2.5 (1‒5)	<0.0001	81.0%	50.0%	55.7%	77.1%
Placental Bulge	4 (1‒5)	3 (1‒5)	<0.0001	95.2%	37.0%	54.1%	90.9%
Heterogeneity	4 (1‒5)	2 (1‒5)	<0.0001	78.6%	55.6%	57.9%	76.9%
Myometrial thinning	5 (1‒5)	3 (1‒5)	<0.0001	95.2%	44.4%	57.1%	92.3%
Placental protrusion sign	2 (1‒5)	1 (1‒4)	<0.001	33.3%	90.7%	73.7%	63.6%
**Radiologist B**							
Intraplacental T2 dark band	4.5 (1‒5)	1 (1‒5)	<0.0001	76.2%	75.9%	71.1%	80.4%
Abnormal vascularity	5 (1‒5)	2 (1‒5)	<0.0001	81.0%	63.0%	63.0%	81.0%
Placental Bulge	4 (1‒5)	2 (1‒4)	<0.0001	85.7%	72.2%	70.6%	86.7%
Heterogeneity	4 (1‒5)	2 (1‒4)	<0.0001	81.0%	57.4%	59.6%	79.5%
Myometrial thinning	4 (2‒5)	3 (1‒5)	<0.0001	83.3%	72.2%	70.0%	84.8%
Placental protrusion sign	2 (1‒5)	1 (1‒4)	<0.0001	45.2%	96.3%	90.5%	69.3%

Min, minimum; max, maximum; SEN, Sensitivity; SPE, Specificity; PPV, Positive Predictive Value; NPV, Negative Predictive Value.

^a^Mann-Whitney test.

The Sensitivity (SEN), Specificity (SPE), Positive Predictive Value (PPV), and Negative Predictive Value (NPV) for each MRI sign are shown in (Table 3).

### Interobserver agreement

Concordance coefficient values are shown in (Table 4). Intraplacental T2 dark band had a substantial agreement between reader A and reader B, moderate agreement for abnormal vascularity and heterogeneity, poor agreement for placental bulge and myometrial thinning, and slight agreement for placental protrusion sign.

**Table 4:** Interobserver agreement.

MRI signs	Radiologists A and B (Kappa coefficient)
Intraplacental T2 dark band	0.749
Abnormal vascularity	0.547
Placental bulge	0.332
Heterogeneity	0.482
Myometrial thinning	0.363
Placental protrusion sign	0.179

A κ-value of ≤ 0.20 was interpreted as slight agreement, 0.21–0.40 fair agreement, 0.41–0.60 as moderate agreement, 0.61–0.80 as substantial agreement and ≥ 0.81 as almost perfect agreement.

### Predictor model

Initially, in other to create a reproducible predictor model, the authors excluded the MRI signs that showed a slight agreement (κ-value of ≤ 0.20) and fair agreement (κ-value of 0.21–0.40) in the interobserver analysis.

The remaining MRI signs (heterogeneity, abnormal vascularization, and intraplacental T2 dark band) and the most relevant clinical sign (previous C-section) were analyzed using a multiple binary logistic regression model. The most significant clinical variable in isolation was the presence of a previous C-section with an OR = 3.35 (95% CI 1.88‒5.97), and the most significant MRI sign was intraplacental T2 dark band with an OR = 12.67 (95% CI 3.97‒40.45) as shown in Table 5.

**Table 5:** Multiple binary logistic regression models of independently most significant variables.

Variables	Coefficient	SE	OR_adj_	95% IC (inf-sup)	p
**Initial analysis**					
Intercept	-3.1	0.6			
Heterogeneity	-1.0	1.1	0.3	0.04‒3.4	0.373
Abnormal vascularity	0.7	0.6	2.0	0.5‒7.4	0.266
Intraplacental dark T2 band	2.9	1.2	19.4	1.8‒210	0.014
Previous C-sections	1.2	0.3	3.4	1.8‒5.9	<0.0001
**Final analysis**					
Intercept	-3.1	0.6			
Intraplacental dark T2 band	2.5	0.6	12.6	3.9‒40.4	<0.0001
Previous C-sections	1.2	0.3	3.4	1.8‒5.9	<0.0001

SE, Standard Error; adj, adjusted; inf, inferior; Sup, Superior; p<0.05.

**Fig. 6:**
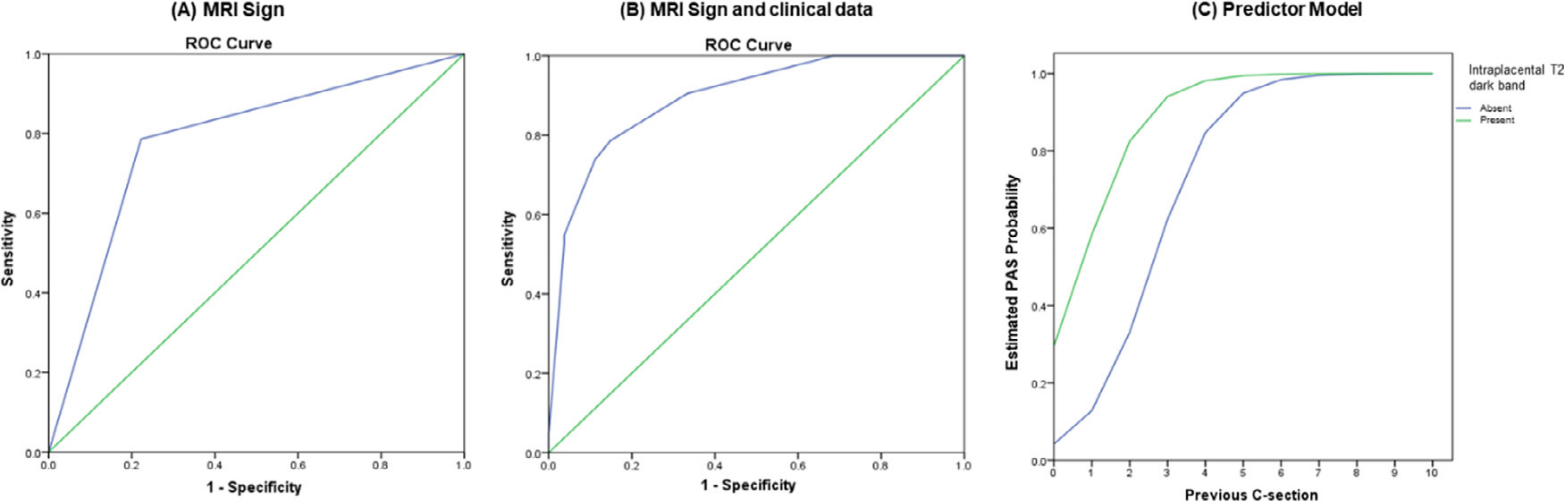
(A) ROC curve analyses for the prediction of PAS including only MRI sign (intraplacental T2 dark band) with an AUC=0.782 (95% CI 0.685‒0.878) and (B) including MRI sign and relevant clinical data (previous C-sections) with an AUC=0.893 (95% CI 0.829‒0.957). (C) Predictor model for the estimated PAS probability versus number of previous C-sections of the patients.

The result of ROC analyses for the prediction of PAS using only the most significant MRI sign (intraplacental T2 dark band) and using the most significant MRI sign combined with the most significant clinical data (previous C-sections) are shown in Fig. 6. For the prediction using only the most significant MRI sign, the area under the curve is 0.782 (95% CI 0.685‒0.878), and for the prediction of the most significant MRI sign combined with previous C-sections, the area under the curve is 0.893 (95% CI 0.829‒0.957).

Finally, the authors derived a predictor model for the estimated PAS probability according to the number of previous C-sections for patients that had or not the presence of the intraplacental T2 dark band sign (Fig. 6).

## Discussion

The analysis in the present study has confirmed that MRI signs of PAS (intraplacental abnormal vascularity, Intraplacental T2 dark band, placental bulge, placental protrusion sign, myometrial thinning, and heterogeneous placenta) were associated with the presence of PAS for both inexperienced and experienced radiologists as observed in previously published studies.

The authors have also evaluated the interobserver variability of MRI analyses of experienced and less experienced radiologists, knowing that the previous experience of the radiologist improves the diagnostic performance, as shown by Ghezzi et al.[34] The authors found a substantial agreement for the intraplacental T2 dark band and a moderate agreement for abnormal vascularity, similarly to previous reports.[19,21,22,28,35,36] The heterogeneity sign also had moderate agreement even though the authors understand that this finding is very influenced by subjective evaluation as resembled on previous studies,[22,28] and it is known that normal placenta can show some heterogeneity.[37] The placental bulge had a fair agreement, a variability slightly higher than previously reported.[19,22,35] Myometrial thinning also had fair agreement similar to what was reported by Lax et al.[19] and in correlation with the knowledge that normal myometrium can become thin during pregnancy, especially in the third trimester and when the placenta has a posterior location.[19,20,27] Placental protrusion sign had a slight agreement and had a low incidence as previously reported by Bourgioti et al.,[36] fact that can explain its low agreement and limit its use once it is a rare finding in a rare disease, making it a difficult sign to be studied and correctly interpreted. These findings indicate the need for objective standardization of MRI assessment of PAS disorders that can provide a more reproductive interpretation of the exam[17] as suggested by the consensus of Jha et al.[38] and potentially reduce the effect of the radiologists' previous experience.[34]

Regarding clinical variables, they can be important risk factors for the development of PAS, mainly in relation to the previous C-section, as previously shown.^4^, ^5^, ^6^ In the present study, radiologists were blinded for clinical information, but the authors understand that the availability of this information to the radiologist at the moment of the exam evaluation is extremely important for the elaboration of his final diagnosis.

The predictive model, including only MRI sing (intraplacental T2 dark band), had great accuracy (AUC = 0.782), which was further improved (AUC = 0.893) when adding previous C-sections. These results demonstrate that this model can be useful for obstetricians to evaluate the estimated probability of PAS on delivery very easily so that they can properly manage it in advance and are not surprised with a potential high-risk procedure.

There are some limitations of the present study. To begin with, it was performed in a tertiary hospital with high-risk patients selected from abnormal ultrasound, inducing selection bias. Moreover, radiologists in the study were well trained for this rare condition, given the particularity of the hospital, and may not reflect standard radiology practice. Some of the advantages of the study are its high number of patients included compared with previous studies and the use of a gold standard combining surgical description and pathological analyses. According to the sample, the results must be analyzed carefully. The reduced sample size contributes to reproducibility issues, including false positives and false negatives. However, the model was analyzed considering the number of outcomes to avoid an overfitting model.

## Conclusion

Simplified objective criteria on MRI (intraplacental T2 dark band) combined with clinical data (previous C-sections) contributed to the creation of a predictive model, which the authors believe can facilitate and improve the diagnostic accuracy by providing a more objective result for the MRI report. Besides, it allows a higher uniformization of the analyses between radiologists with different levels of expertise and facilitates information to obstetricians. Based on these findings, the authors suggest the application of this model in prospective studies in order to elucidate external validation issues.

### Authors' contributions

Rodrigo Pamplona Polizio: Conceptualization, methodology, data collecting, writing-original draft.

Fernando Ide Yamauchi: Conceptualization, methodology, data collecting, review the final version.

Renata Franco Pimentel Mendes: Data collecting, review the final version.

Stela Verzinhasse Peres: Formal analysis.

Mario Macoto Kondo: Review the final version.

Rossana Pulcineli Vieira Francisco: Conceptualization, metodology, writing-review & editing.

## Conflicts of interest

The authors declare no conflicts of interest.
